# Multisystem Inflammatory Syndrome in Children and COVID-19 Infections

**DOI:** 10.1177/1942602X211021136

**Published:** 2021-11

**Authors:** Margaret W. Bultas, Kelli Fuller

**Affiliations:** St. Louis, MO; St. Louis, MO

**Keywords:** COVID-19, multisystem inflammatory syndrome, pathophysiology, school nurse, children

## Abstract

The arrival of SARS-Co-V-2 (severe acute respiratory syndrome coronavirus 2) has brought not only the COVID-19 (coronavirus disease 2019) pandemic but also the development of a cluster of symptoms known as multisystem inflammatory syndrome in children (MIS-C). Information regarding the long-term implications of COVID-19 infections in children, as well as MIS-C, is scarce and is emerging on an almost daily basis. The purpose of this article is to provide an overview of the recent literature regarding COVID-19 and MIS-C, a Kawasaki-like inflammatory syndrome that developed in children around the same time the COVID-19 pandemic began. Because the school nurse monitors children across a variety of developmental domains, they are in a unique position to identify changes and notice long-term trends related to the health and development of children who contract both COVID-19 and MIS-C.

## Introduction

Although the coronavirus disease 2019 (COVID-19) caused by the SARS-CoV-2 (severe acute respiratory syndrome coronavirus 2) virus has globally infected millions of people, serious outcomes in children have been limited thus far; however, this could change as new strains of the virus emerge (Alsaied et al., 2021). Data show infection rates in children less than 18 years of age have been about half of the infection rate in adults, manifestations in children generally less severe, and many with asymptomatic presentations (Alsaied et al., 2021). The medical community has seen an increase in a cluster of concerning symptoms affecting children since the start of the COVID-19 pandemic. This collection of symptoms, now referred to as multisystem inflammatory syndrome in children (MIS-C), was initially thought to be Kawasaki disease (KD) as there are some similarities. However, it has since been determined to be a unique collection of symptoms related to COVID-19 infection (Rowley, 2020). To understand more about MIS-C, it is helpful to understand COVID-19 and review the features of KD. The purpose of this article is to provide information to the school nurse related to the pathophysiology, clinical presentation, and clinical course of COVID-19, KD, and MIS-C as they have overlapping presentations and may have long-term implications in children.

## COVID-19 in Children

In January 2020, the World Health Organization (WHO) declared the novel coronavirus outbreak a Public Health Emergency of International Concern, which is the WHO’s highest level of concern (WHO, 2020). COVID-19 is caused by SARS-CoV-2, which is the seventh coronavirus to infect humans (Ludvigsson, 2020). In general, information related to COVID-19 infections in children has been limited, as children have been less severely affected by the initial strains of COVID-19 infections (Ludvigsson, 2020; Rowley, 2020). However, infants less than 1 year of age and children with comorbidities do tend to exhibit more serious disease and present with acute respiratory distress syndrome and multiorgan failure similar to adults (Alsaied et al., 2021; Centers for Disease Control and Prevention [CDC], 2021a). It is suspected children most often contracted COVID-19 within their family cluster (Ludvigsson, 2020). Comorbidities that may lead to more severe COVID-19 infection in children include asthma/chronic lung disease, diabetes, neurological and metabolic conditions, sickle-cell disease, congenital heart disease, obesity, a compromised immune system, and children with complex medical conditions (CDC, 2021a, 2021c).

Although children tend to present with less severe symptoms, those who do develop symptoms most often present with fever, cough, and pharyngeal erythema (Ludvigsson, 2020). Other symptoms may include runny nose, loss of taste/smell, sore throat, difficulty breathing, diarrhea, malaise, headache, body aches, and poor feeding in infants (CDC, 2021a). However, there is concern that new, variant mutations of COVID-19 could be more infectious and symptom producing for children than the original strain (Galloway et al., 2021).

Several theories have been proposed as to why children respond with less illness and symptoms, as compared with adults, to COVID-19 infections. One theory is children may have a more robust immune system, due to the frequency of viral infections during childhood, and may be better at responding to SARS-CoV-2 (Ludvigsson, 2020). Another theory is related to the linkage of SARS-Co-V-2 and angiotensin-converting enzyme (ACE; Consiglio et al., 2020). The virus has been shown to bind to ACE2 receptors expressed in the epithelial cells in the lungs, intestines, kidneys, brain, and blood vessels (Bourgonje et al., 2020). In children, ACE is less mature and may provide some level of protection against the virus in children (Ludvigsson, 2020). Last, COVID-19 infection in adults more often results in an exuberant inflammatory response. Inflammatory responses differ and vary across the life span, which may partially explain the decreased severity of the virus in children (Molloy & Bearer, 2020).

## A Review of Kawasaki Disease

KD is a febrile illness involving the inflammation of blood vessels, vasculitis, which can result in coronary artery and cardiovascular complications in young children (Rowley, 2020; Toubiana et al., 2020). KD predominately affects children under the age of 5 years, and the global incidence is highest in Japan and in children of Asian ethnicity (Toubiana et al., 2020). The etiology of KD is not precisely known; however, it is suspected it may be linked to a viral trigger with a seasonal variation (Rowley, 2020). KD has been associated with other coronaviruses (Diorio et al., 2020).

Typical manifestations of KD include fever for more than 5 days, polymorphous rash, inflamed conjunctiva, cervical lymphadenopathy, and redness of the oropharynx (Diorio et al., 2020; Rowley, 2020). The clinical course of KD is significant as the coronary arteries can become dilated with the subsequent development of aneurysms predisposing the child to coronary artery ruptures in 20% of cases (Diorio et al., 2020). The development of shock in KD is less common and affects only about 10% of patients (Diorio et al., 2020).

Treatment of KD includes hospitalization with intravenous infusion of immunoglobulin and aspirin therapy for reduction of inflammation and antiplatelet effects (Soma et al., 2021). Patients may continue aspirin therapy after hospital discharge. Follow-up with cardiology will continue for several months to monitor for the development of coronary artery aneurysm with echocardiograms.

## Multisystem Inflammatory Syndrome in Children

### Etiology and Epidemiology of MIS-C

During the COVID-19 pandemic, a collection of symptoms appeared to simultaneously develop in children. Initially thought to be KD, due to the similarity of some inflammatory symptoms and cardiovascular involvement, differences in the presentation become clearer and it was named MIS-C. This new syndrome presented a range of clinical symptoms temporal to COVID-19 infections and appeared 4 to 6 weeks after COVID-19 infection or exposure (Consiglio et al., 2020; Feldstein et al., 2020; Rowley, 2020).

**Figure 1.**
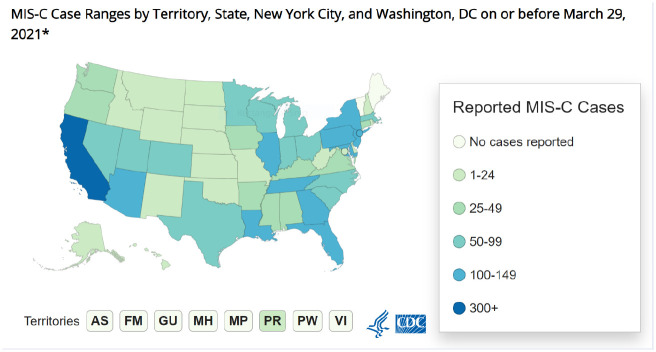
Case Map From the Centers for Disease Control and Prevention *Note*. Accessed April 21, 2021, from the Centers for Disease Control and Prevention. https://www.cdc.gov/mis-c/cases/index.html.

Because of the timing, the etiology is suggestive of a link to the SARS-CoV-2. MIS-C primarily affects older children and adolescents (CDC, 2021b; Rowley, 2020). Initial case reports of MIS-C were seen in Italy, the United Kingdom, and areas of the United States that were experiencing large outbreaks of COVID-19 (Soma et al., 2021); however, few to no cases of MIS-C were reported in China and Japan (Rowley, 2020). The CDC continues to track and compile data on MIS-C and has identified more than 3,180 cases in the United States as of March 2021. Ninety-nine percent of cases were positive for SARS-Co-V-2, and those who were not positive were exposed to someone who was positive (CDC, 2021b). The highest incidence of MIS-C cases occurred in California, New York City, New Jersey, Illinois, Tennessee, Louisiana, Georgia, and Florida, which all had higher COVID-19 infection rates in the early months of the pandemic as shown in Figure 1 (CDC, 2021b). Data show that as COVID-19 infection rates decreased, so have the rates of MIS-C (CDC, 2021b). The age of those affected by MIS-C, in the United States range from infancy to 20 years of age, with a median age of 9 years, and most cases occurring in the 1- to 14-year age-group (CDC, 2021b). Of the cases in the United States, 66% occurred in children who are Hispanic/Latino or Black, non-Hispanic, and more than half (59%) were male (CDC, 2021b; Jiang et al., 2020; Soma et al., 2021). European countries also report similar patterns with an increase in cases of MIS-C in those with African ethnicity (Ahmed et al., 2020).

### Manifestations and Clinical Course of MIS-C

**Figure 2.**
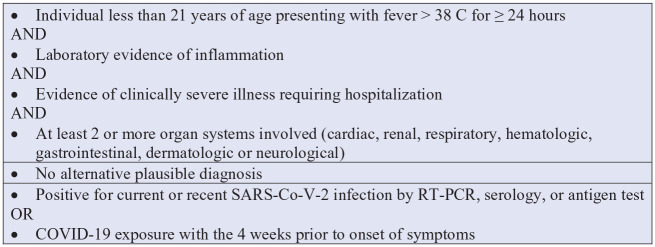
CDC Case Definition for MIS-C *Note*. Criteria are met if all three boxes are met (CDC, 2021b). CDC = Centers for Disease Control and Prevention; MIS-C = multisystem inflammatory syndrome in children.

Children with MIS-C most commonly presented with gastrointestinal symptoms and abdominal pain, fever, multiple organ involvement, and shock often requiring inotropic and vasoactive support and care in the intensive care unit (Feldstein et al., 2020; Toubiana et al., 2020; Soma et al., 2021). Other manifestations include hematologic effects with increased inflammatory markers, mucocutaneous symptoms of red eyes and pharynx, and respiratory symptoms (Feldstein et al. 2020; Rowley, 2020). Patients had an average of four organ systems involved with the most effected being the gastrointestinal, cardiovascular, hematologic, mucocutaneous, and respiratory systems (Feldstein et al., 2020). Approximately 22% of MIS-C patients experience neurological manifestations, such as seizure, stroke, and encephalopathy (LaRovere et al., 2021). The CDC has defined MIS-C using a specific set of criteria as listed in Figure 2 (CDC, 2021b).

Clinical course for MIS-C included approximately 1 week in the hospital, recovery within a couple of weeks, and overall low mortality rates (Alsaied et al., 2021). About 80% of patients required intensive care unit support for intravenous vasoactive therapy for shock symptoms with a smaller portion also requiring ventilation (Feldstein et al., 2020). Most patients experienced left ventricular dysfunction with decreased ejection fractions, myocarditis, and arrythmias in the acute period of MIS-C, which appeared to resolve after several weeks (Alsaied et al., 2021).

### Pathophysiology of MIS-C

Although the pathophysiology of MIS-C is not completely known or understood, the syndrome appears to be an inflammatory response to the SARS-CoV-2 pathogen involving multiple autoantibodies (Consiglio et al., 2020). It has not been determined if MIS-C is an ongoing infection with COVID-19 or (more likely) a postinfection immune response (Soma et al., 2021). Considering children and adults have differing inflammatory and immune responses, it has also been hypothesized children with MIS-C may be experiencing a slower viral clearance or delayed inflammatory response—the reason why the syndrome appears several weeks after COVID-19 infection or exposure (Rowley, 2020). Other research has shown that children with MIS-C only generated antibodies to the spike protein rather than antibodies to the full virus capsid, which possibly resulted in an inefficient or altered immune response in those individuals (Soma et al., 2021).

Inflammatory lab markers in children with MIS-C appear to be of a different pattern than in adults with COVID-19 and those with KD. In general, MIS-C is associated with increased interleukin (IL) levels for IL-6, neutrophils, troponin, brain natriuretic peptide, C-reactive protein, increased sedimentation rates, and altered T cell counts (Ahmed et al., 2020; Cattalini et al., 2021; Feldstein et al., 2020; Rowley, 2020; Soma et al, 2021). Initial research points to children who present with higher neutrophil to lymphocyte ratios tended to experience more life-threatening neurological conditions (LaRovere et al., 2021). It should be noted that there are no specific set of biomarkers that have been validated for MIS-C, however.

Some children present with the vasculitis of the coronary arteries, fever, adenopathy, and the mucocutaneous findings that are similar to those in KD. However, MIS-C more frequently presents with gastrointestinal symptoms, hypotension, and left ventricular dysfunction (Soma et al., 2021). Epidemiologically, MIS-C cases are affecting older children with few cases being reported in Japan, China, and Korea, which have the highest number of KD cases (Soma et al., 2021).

### Treatment and Ongoing Care for MIS-C

Since many features of MIS-C have similarities with KD, treatments overlap. It should also be noted that limited evidence and research exist on treatment for MIS-C.

Treatments during the acute period include intravenous immunoglobulin and glucocorticoids to manage the inflammatory response and aspirin therapy to prevent platelet aggregation in cases of coronary artery enlargement (Cattalini et al., 2021). Most children respond well to these anti-inflammatory therapies, which supports the underlying theoretical basis of inappropriate immune activation (Soma et al., 2021). Biologic agents and antibiotics may also be used on a limited basis when indicated. Unique to MIS-C, vasopressors and inotropic support are commonly used due to the left ventricular dysfunction and signs of shock.

Children are to be discharged from the acute care setting once inflammatory markers have normalized, fever has resolved, blood pressure has normalized, the child does not require oxygen support, and the child is well hydrated (Jiang et al., 2020). Long-term follow-up for children recovering may include the following teams: infectious disease, rheumatology, cardiology, neurology, and hematology. Because long-term outcomes, especially related to cardiovascular status, are not known, children may be followed at intervals over time (Jiang et al., 2020).

## Implications for School Nurses

Due to the novel nature of MIS-C, there is still much to learn. Given MIS-C develops 4 to 6 weeks after a COVID-19 infection or exposure, school nurses are in a position where they may be one of the first health providers to identify symptoms of MIS-C. Students who present with fever, gastrointestinal symptoms, and new onset of activity intolerance related to cardiovascular effects, after a COVID-19 infection or exposure, warrant additional concern. Students recovering from MIS-C also merit a watchful eye as little data on the long-term outcomes are known. Cardiovascular complications, for example, may first be identified by the school nurse as new activity intolerance. Additionally, school nurses may recognize cognitive, developmental, and social changes, which could indicate ongoing neurological concerns (LaRovere et al., 2021). Students with ongoing health concerns may benefit from a 504 plan.

Regarding COVID-19 infections, even though initial variants have resulted in milder cases in children and adolescents, information about the infectiousness and manifestations of new variants is not well-known. The CDC continues to provide updated guidance as new information develops; this can be accessed at https://www.cdc.gov/coronavirus/2019-nCoV/index.html. School nurses should regularly check the CDC COVID-19 website for developments related to MIS-C, COVID-19, and long-term effects of post-COVID infections as information continues to develop. Contact tracing should also continue as new variants emerge, and these may result in different manifestations and outcomes in children. Current criteria for isolation for those who contracted the virus includes the following: 10 days since the first symptoms have appeared, a period of 24 hours where there has been no fever, and other symptoms are improving (CDC, 2021a). The CDC also provides a helpful guideline called *Back to School Planning Checklist for Parents, Caregivers, and Guardians* (CDC, 2021a).

## Conclusion

Information regarding COVID-19 infections and MIS-C in children continues to develop as new information is learned. School nurses are in an important position to notice and identify trends related to long term effects and outcomes of COVID-19 infections in children. School provides the opportunity to monitor cognitive, physical, and social development of children, and nurses are on the front lines in identifying trends, long term concerns, and educating parents on important aspects of COVID-19 infections including prevention and treatment. ■
